# Evaluation of high resolution thermal imaging to determine the effect of vertebral fractures on associated skin surface temperature in children with osteogenesis imperfecta

**DOI:** 10.1007/s11517-018-1806-3

**Published:** 2018-02-26

**Authors:** Alexandra Fane De Salis, Reza Saatchi, Paul Dimitri

**Affiliations:** 1 The University of Sheffield, Sheffield, UK; 2 Sheffield Hallam University, Sheffield, UK; 3 Sheffield Children’s NHS Foundation Trust, Sheffield, UK

**Keywords:** Computerised medical diagnosis, Thermal imaging, Vertebral fracture detection, Osteogenesis imperfecta

## Abstract

Vertebral fractures are common in children with osteogenesis imperfecta (OI). Current imaging methods for fracture detection (X-ray and DXA) use ionising radiation. This pilot study explored whether the alteration in blood flow in vertebral fractures results in skin temperature changes that may be detected using high resolution thermal imaging (HRTI) and thus assist diagnosis and monitoring of fractures in OI patients. Eleven participants aged 5–18 years with OI and known vertebral fractures were enrolled. Small metal discs were placed on the skin surface alongside the vertebrae before participants had DXA and X-ray scans and thermal imaging of their backs. Visibility of the discs on the DXA and X-ray scans and thermal images allowed the temperatures of the skin surface above vertebrae without (healthy) and with fractures to be compared to their respective adjacent skin surface regions (region of reference, ROR) by calculating the temperature percentage change (TPC). The TPC between the skin temperature over the fractured thoracic vertebrae (*n* = 11) and the ROR was significant (1.44%, *p* = 0.002, 95% confidence). TPC between the skin temperature over healthy thoracic vertebrae and ROR was not significant (0.97%, *p* = 0.15, 95% confidence). HRTI may provide a novel tool for assisting in detection of vertebral fractures in OI.

**Graphical abstract:**
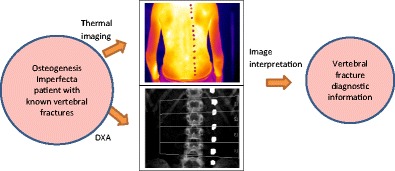
• Patients (aged 5–18) with osteogenesis imperfecta and known vertebral fractures. • Thermal imaging was performed alongside routine imaging (DXA scan and spinal X-ray). • The temperature above each vertebra was compared with its adjacent skin region to assist with diagnosis of the fracture.

• Patients (aged 5–18) with osteogenesis imperfecta and known vertebral fractures.

• Thermal imaging was performed alongside routine imaging (DXA scan and spinal X-ray).

• The temperature above each vertebra was compared with its adjacent skin region to assist with diagnosis of the fracture.

## Introduction

Thermal imaging (TI) has been studied in the medical field since the 1960s. It measures the infrared (IR—wavelength 0.8–1000 μm) emissivity of objects and provides a quantitative and qualitative map of the temperature distribution in real time 2D images or 3D data [1]. This is achieved by detectors that transform the infrared radiation into temperature values represented as a digital image [2]. TI has been used in the medical field to examine temperature anomalies in breast cancer, infections, and eye conditions [3]. In paediatrics, TI has been used to determine therapeutic change in haemangioma and vascular malformations and has potential to identify musculoskeletal injuries [3]. Furthermore, the use of TI for monitoring sleep apnoea [4], sleep dynamics [5] and as a non-contact method to measure respiration rate has been reported [6]. In each application, the alteration in blood flow provided a ‘relative’ temperature change [7]. However, further TI technological improvements and image processing developments were needed to translate the use of TI technology in routine clinical practice. Current TI cameras can detect much smaller differences in temperature, are more affordable and there have been extensive advances in thermal image processing. Therefore, the applications of TI in medicine should be re-evaluated given the new opportunities it can provide as a non-invasive and non-ionising diagnosis and monitoring tool.

**Fig. 1:**
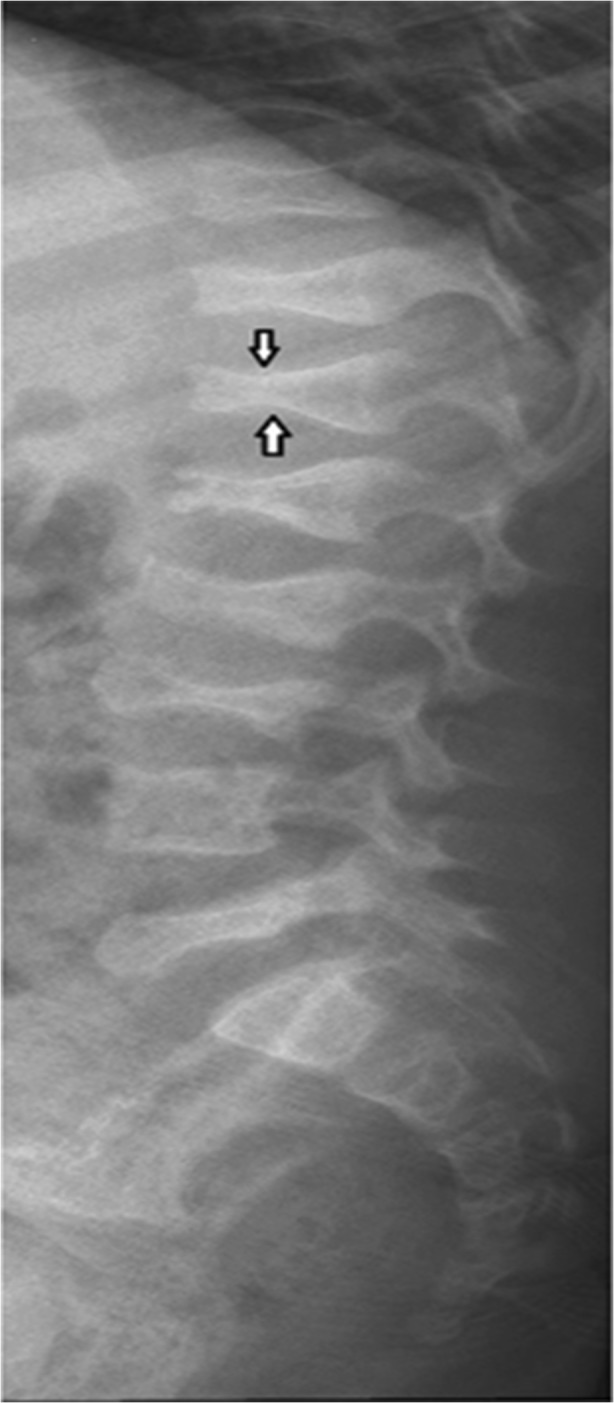
Lumbar and thoracic spine radiograph of a 6-year-old child with OI demonstrating anterior wedge compression fractures at multiple vertebrae [17]

Objects with temperatures greater than 0 degree Kelvin (− 273 °C) emit infrared radiation (IR) [8]. The skin is a highly efficient IR emitter with an emissivity (ε) of 0.97 [9] as it emits heat from its surface to regulate body temperature whilst the core body temperature acts a thermal reservoir and stays relatively stable [8, 10]. Alterations in skin surface temperature relate to variations in the blood flow or inflammation and provide a means by which the temperature changes could be used for medical monitoring and diagnosis. Thermal imaging of the skin surface is performed in long-wavelength IR (LWIR) (8–14 μm) because emissivity is highest in this band and the IR radiation is less attenuated by the atmosphere [11]. An area of potential application for TI in the medical field is in the change in blood flow relating to bone fractures. Blood flow within a fracture site changes rapidly following a fracture and combined with the inflammatory response and will result in a change in thermal output that in theory could be detected if the change is sufficiently strong and close to the skin surface. Patients with osteogenesis imperfecta (OI, also known as brittle bone disease) are at high risk of bone fracture, bone deformity and growth deficiency as a result of low bone mass and increased bone fragility [12] caused by a mutation in the type I collagen gene. OI has been classified into four types (I–IV) depending on clinical presentation and sub-grouped by radiographic findings [12]. Thus, due to recurrent fractures, children with OI can be frequently exposed to ionising radiation from bone densitometry scan (DXA) and X-ray radiograph. In OI, vertebral fractures can present as back pain or scoliosis but many are clinically silent and only detectable by spinal X-ray radiographs and vertebral fracture analysis (VFA) using DXA [13, 14]. A vertebral fracture is seen on spinal X-ray radiographs as a change in the morphology of the vertebral body with a decrease in more than 20% of vertebral height and classified using the Genant semiquantitative (GSQ) method [15–17] (see Fig. 1).

Early identification and subsequent treatment of vertebral fractures are necessary to prevent spinal deformities [18]. Following a fracture, bone healing occurs in three phases: inflammatory, reparative and remodelling. A temperature change occurs during each phase as the blood flow to the fracture site is altered, an inflammatory response develops and the bone re-models over a period of months.

Our pilot study aimed to develop a novel methodology based on high resolution thermal imaging (HRTI) to explore whether possible alterations in the blood flow following vertebral fractures in children with OI could lead to a significant change in the skin surface temperature over the fractured vertebrae that may be detected by HRTI and thus assist in screening for vertebrae fractures.

## Methodology

### Recording procedure

**Fig. 2:**
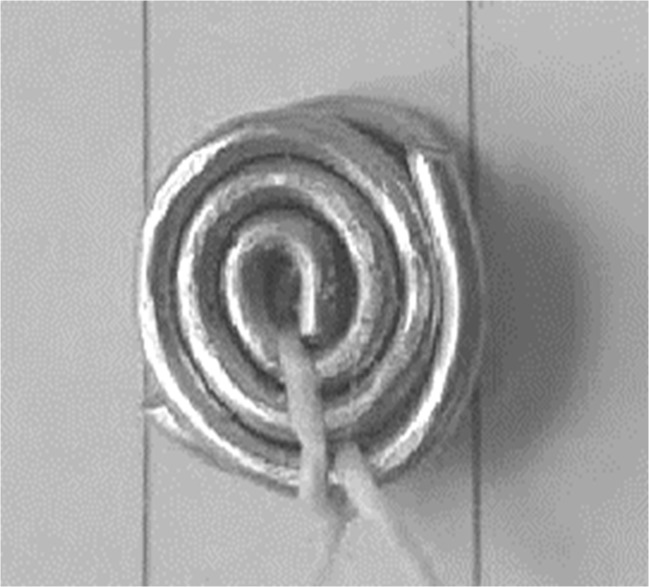
A typical steel disc used in the study

Ethical approval for the study was obtained from the National Research Ethics Service (UK-REC reference 15/NW/0770). Eleven consented patients aged 5–18 years (standard deviation = 4.0 years) with osteogenesis imperfecta (OI) and at least one previously identified vertebral fracture were recruited from the OI service at Sheffield Children’s NHS Foundation Trust, UK. Only the patients that were due to have a DXA scan or spinal X-ray as part of their clinical care during the study period were included in the study. All patients were receiving bisphosphonates as part of their medical treatment. Prior to DXA scans or spinal X-ray radiograph, patients had small steel discs placed on their back to mark the approximate positions of vertebrae. These radiopaque discs were constructed from two 1-mm-thick steel wires twisted into tight circular rings with a diameter between 7.5 and 8.5 mm and held together with string as shown in Fig. 2 (two discs were used as a single disc was not sufficiently visible in the DXA and X-ray radiograph scans).

**Fig. 3:**
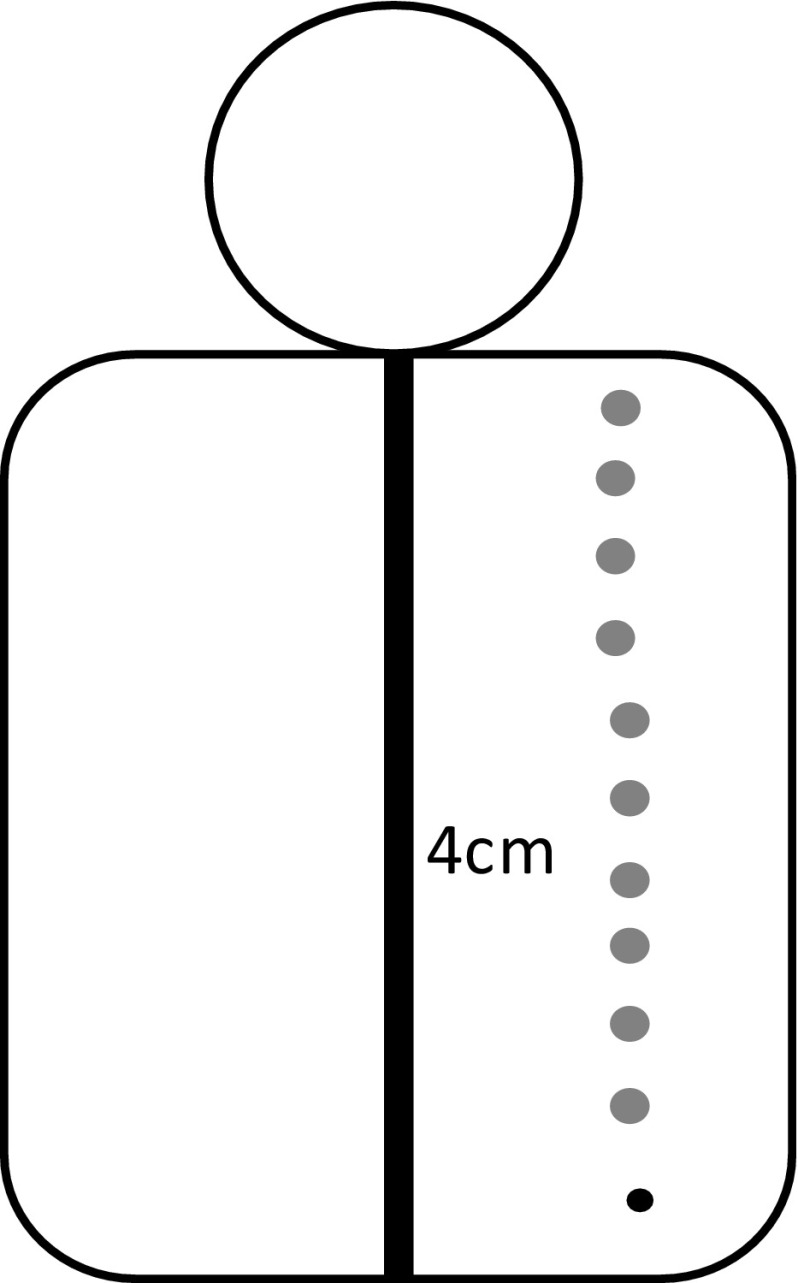
The position of the metal discs on a patient’s back. Metal discs (grey circles) positioned 4 cm to the right of each vertebra (black vertical line). The black circle denotes the position of the lowest vertebra before the disc was stuck on

This design was used to ensure the discs were sufficiently light to remain attached to the patient’s back and to be dense enough to be visible on the thermal, DXA and X-ray radiograph images. The discs were attached to the patient’s back using a double-sided tape, 4 cm to the right of the spine and parallel to each spinous process identified by palpation (Fig. 3).

A felt-tip pen was used to mark the discs’ locations so they could be replaced if they fell. The discs were visible on the DXA, X-ray radiograph and thermal images and thus provided an effective guidance to allow for visual comparison of the vertebral positions on the DXA, X-ray radiographs and thermal images.

As part of their clinical management, the patients with OI have DXA scans every 6 months and spinal X-ray radiograph scans annually. Therefore, only five of the patients had both their X-ray radiographs and DXA scans taken during the time of the study. Following the DXA or X-ray radiograph, the patients underwent thermal imaging of their exposed backs. The room used for thermal imaging did not have active central heating radiators and was not prone to drought. The temperature and humidity of the room were measured prior to each thermal image recording using a HTC-1 Digital LCD Temperature Humidity Meter. Window blinds were closed to minimise environmental temperature influence and the participants were positioned in the corner of the recording room as far away from the door and window as possible. The number of people in the recording room was kept to a minimum. The patients sat on stool with their upper body exposed for 10 min to acclimatise to the room temperature. The 10 min was a compromise between allowing sufficient acclimatisation of the patients and ensuring they did not get tired (given the patients were children and young people). We also aimed to make the experimental methodology as realistic as possible to the manner it could realistically be applied in clinical diagnostic situations.

**Fig. 4:**
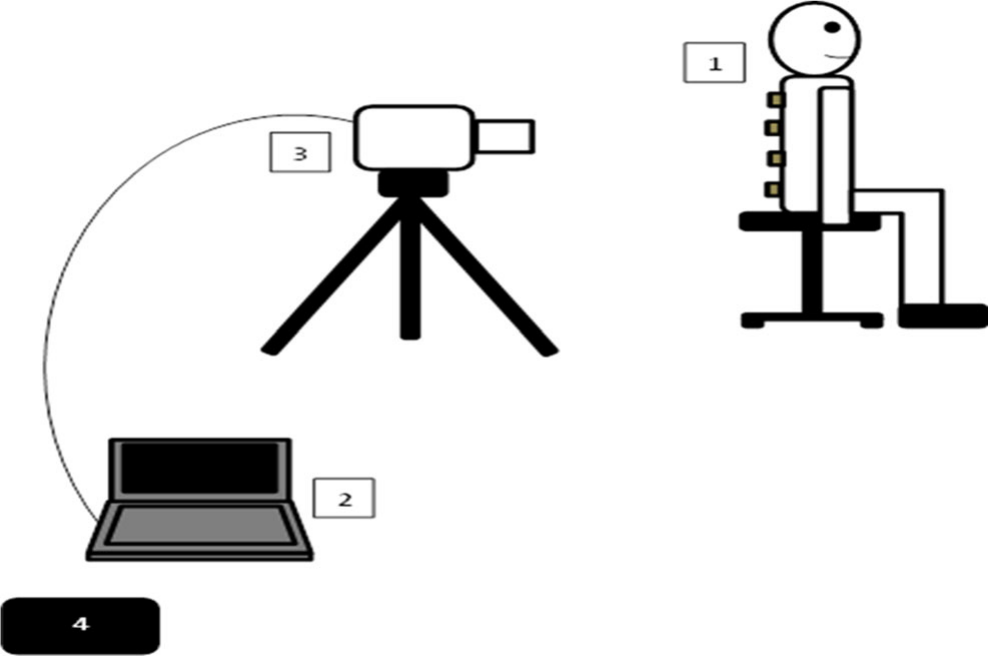
A set up for data recording. (1) patient (the grey squares represent the steel discs placed on the patient’s back, discs not to scale and their number is for illustration), (2) laptop computer, (3) thermal camera, (4) temperature and humidity meter.

A calibrated FLIR® T630sc infrared camera was used for the thermal imaging. The camera has an image resolution of 640×480 pixels, spectral range of 7.5–13 μm, temperature sensitivity within 0.04 m-Kelvin, maximum image capture rate at full resolution 30 frames per second and standard temperature measurement range of − 40 to 650 °C. The camera’s emissivity value was set to 0.97 as this is suitable for human skin temperature measurements. The image capture rate was set to the maximum 30 frames per second (this rate was considered sufficient as there was minimal body movement). The camera was fully calibrated prior to the commencement of the study. The camera was mounted on a lightweight carbon fibre tripod (Manfrotto, Italy) that allowed its height and angle to be accurately adjusted. The height of the tripod and its distance from the participant was adjusted for each recording so that the camera was perpendicular to the participant’s back and the whole back was in the camera’s field of view. The camera distance to the participant was set at 1 m (Fig. 4).

**Fig. 5:**
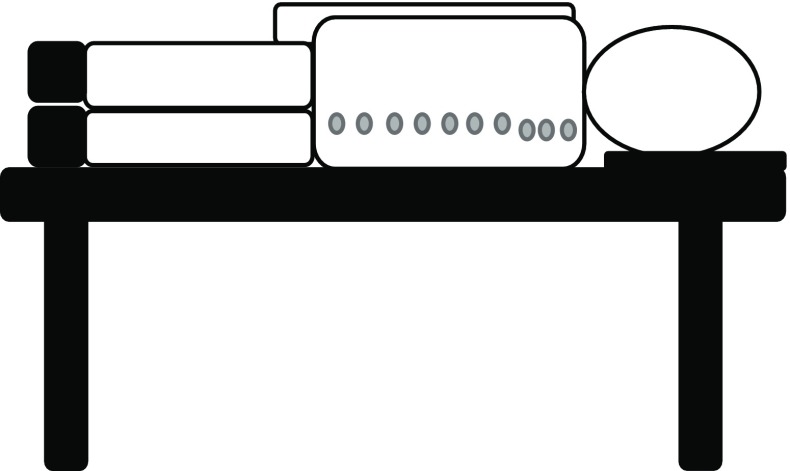
Alternative recording position when the patient was unable to sit unaided for 12 min. The steel discs are depicted by grey circles (not to scale)

The patients who were unable to sit upright independently due to the severity of their medical condition lied on their side as illustrated in Fig. 5. The duration of each thermal video recording was 2 min providing 3600 frames (i.e. 2 ×60 ×30). During the recordings, there were small involuntary body movements caused by breathing and restlessness. Therefore, the spine’s position in the successive images changed over time. This however was not significantly large to affect temperature averaging over time and was further minimised by only analysing the first 60 frames (2 s) of recording during which the least movement occurred. Two seconds of recording allowed for at least one heart beat and thus catered for possible temperature fluctuations at the measurement site as blood flowed from and returned to the heart.

All recordings were performed by a single member of the research team to ensure consistency. She had been suitably trained to performed thermography.

### Interpretation of X-ray radiographs and DXA scans

The spinal X-ray radiographs and DXA images were interpreted independently by two researchers, a senior clinician in paediatric musculoskeletal imaging and a trained senior researcher with relevant expertise. They used the SpineAnalyzer™ software to interpret the radiographs. The software assessed the deformity of vertebrae from the vertebrae level T4 (thoracic) to L4 (lumbar) using a six-point morphometry, with points defined by the user. The features available in the picture archiving and communication system (PACS) software were used for the lateral view DXA images. Vertebrae were classified as D0 (normal or healthy) to D3 (with severe fracture) depending on the percentage of vertebral body height lost as indicated in Table 1.

**Table 1:** Vertebral height loss classification

Percentage of vertebral body height lost	Identifier	Vertebral classification
≤ 10.9	D0	Normal (healthy)
11–25.9	D1	Mild fracture
25–49.9	D2	Moderate fracture
≥ 50	D3	Severe fracture

Some vertebrae could not be classified as their details were not accurately measurable on the images. Only the vertebrae that the two experts had fully agreed on their classifications were included in the study and others were excluded.

The thermal image recordings were analysed and compared to the images obtained from DXA and X-ray radiographs using a MATLAB® (version 8.3, R2014a) data analysis package. In cases where both DXA images and spinal X-ray radiographs were taken, an agreement was reached visually on the positions of vertebral fractures identified on both imaging modalities.

### Analysis methodology

**Fig. 6:**
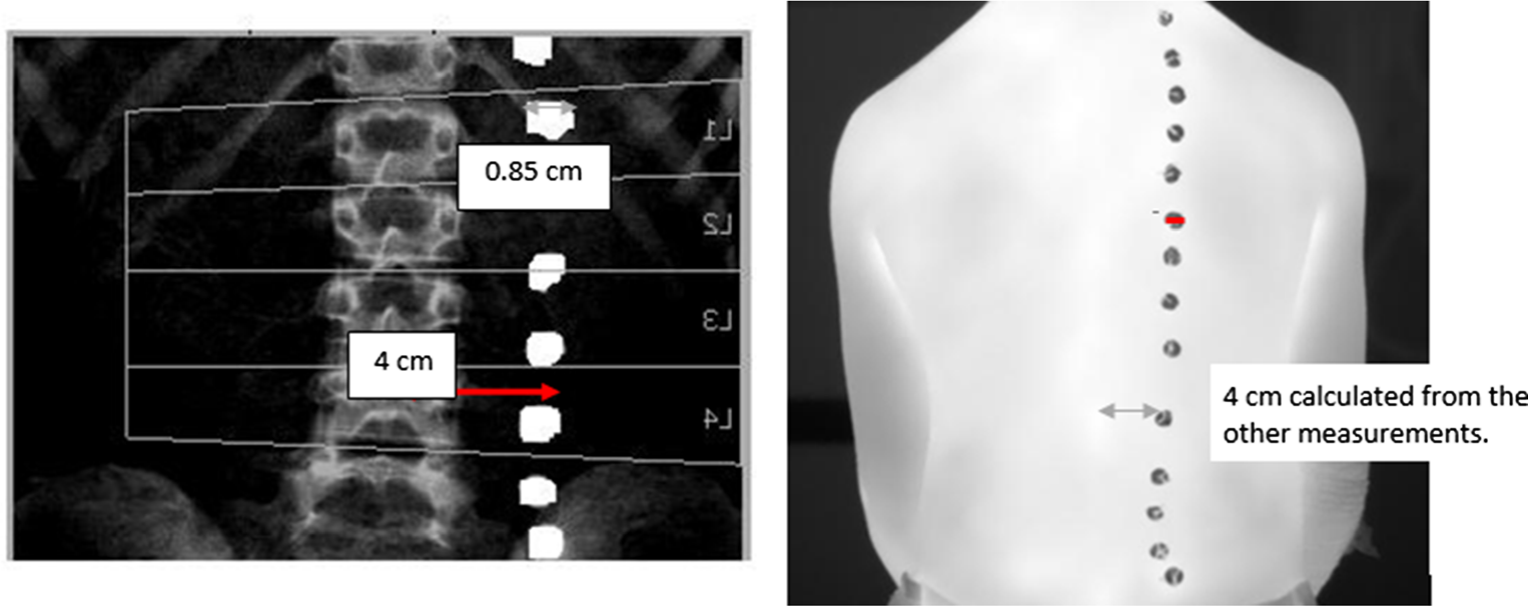
Anteroposterior DXA image that has been flipped horizontally and thermal image showing how the locations of the spinous processes on the thermal image were determined

**Fig. 7:**
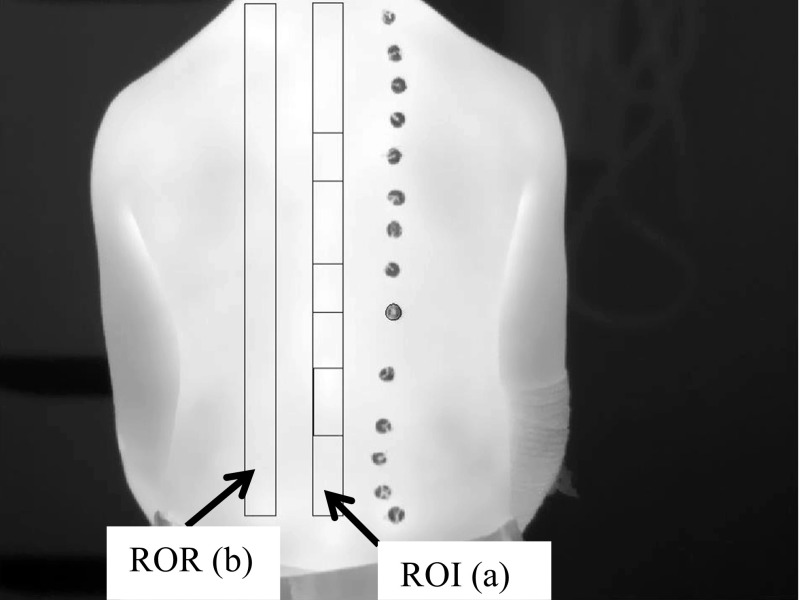
A typical thermal image of a patient’s back. The region of interest (ROI) (**a**) and region of reference ROR (**b**) are shown as plotted in MATLAB®.

MATLAB® data visualisation analysis package was used to display the thermal images alongside the corresponding DXA. For the patients with their spinal X-ray radiographs available, their radiographs were also displayed. The steps to analyse the data are as follows:

- Step 1: The anterior posterior (AP) DXA images were flipped horizontally and compared with the thermal images. Vertebrae were identified on the thermal images by calculating the distance (based on image pixel count) between the centre of each metal disc and its corresponding spinous process (a bony projection off the posterior of each vertebra) on the DXA images and translating this across. The distance between the spinous processes and the centre of the corresponding disc on the DXA image was 4 cm and so a ratio was calculated using pixels to confirm the location of the spinous processes on the thermal image as shown in Fig. 6.

- Step 2: Using the MATLAB’s© graphic user interface facilities, a rectangular region of interest (ROI) was manually positioned over the image of the identified spinous processes to select the spinal column. A comparable region of reference (ROR) was created parallel and to the left of the spine covering the adjacent skin region with the same size and shape as the ROI (Fig. 7).

The distance between the ROI and ROR was 1–2 cm depending on participant’s body size. This gap was introduced to reduce any possible temperature interaction between ROI and ROR whilst ensuring that an accurate comparison could be made between the ROR and ROI. A gap of 1–2 cm was also left between the base of the ROI/ROR and the patient’s trousers to ensure the heating/cooling effect of clothes did not affect the measured temperatures.

**Fig. 8:**
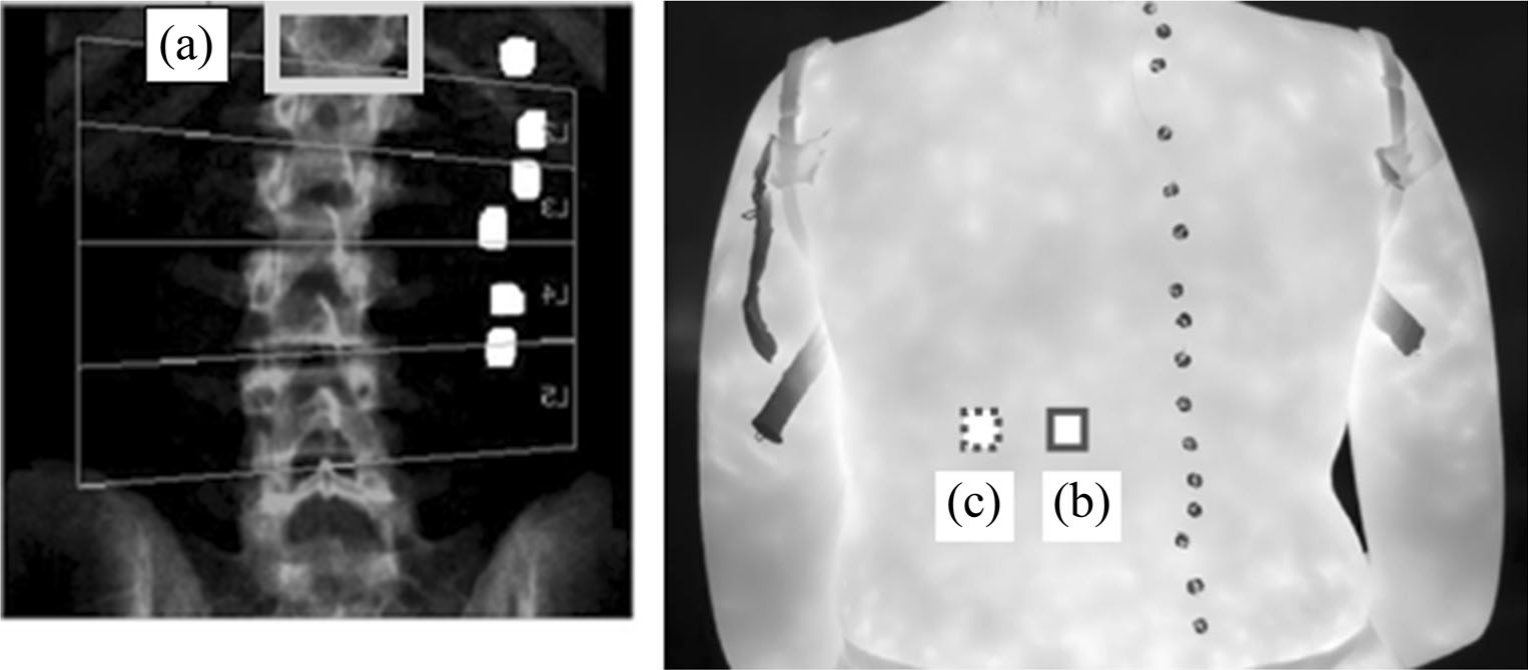
An AP spine DXA image flipped horizontally (left) and thermal image (right). In the DXA image, the T12 vertebra is fractured (light grey box, A) and the corresponding location on the thermal image (dark grey box, B) was correlated by counting the metal discs and observing their pattern. The comparison adjacent reference region is to the left of the dark grey box (B) (dotted grey box, C)

- Step 3: The fractured vertebrae were identified by carefully considering the positions of metal discs on DXA and locating the spinal column (ROI) and corresponding metal discs on the associated thermal image. The ROR was used to provide the area of comparable adjacent skin the same size and shape as the fractured vertebra. When two adjacent vertebrae were fractured, the skin temperature over both vertebrae was measured (Fig. 8).

- Step 4: For a comparable measurement of skin temperatures adjacent to the heathy (i.e. not fractured) vertebrae, their distances were measured in the similar manner to the fractured ones. The healthy vertebrae were chosen to be a number of vertebrae away from the fractured vertebrae to minimise their temperature interactions.

- Step 5: MATLAB® was used to determine the mean temperature for each selected region for each patient. The resulting data were then used to calculate the temperature difference between the selected vertebrae and its adjacent reference skin region by determining the average temperature percentage change (TPC) using Eq.1.

1 $$ TPC=\frac{1}{n}\sum \limits_{i=1}^n\left(\frac{v-r}{r}\right)\times 100 $$

where *v* is the mean skin temperature over the selected (fractured or healthy) vertebra, *r* is the mean skin surface temperature of the region adjacent to the selected vertebra and *n* is the number of vertebrae cases included in the measurements. This was also performed for the selected healthy vertebrae and their respective adjacent reference skin regions.

The TPC determines the percentage temperature change, but positive and negative values cancel each other in its calculation. Therefore, the absolute temperature percentage change (ATPC) was also calculated that ignores the differences in signs as

2 $$ ATPC=\frac{1}{n}\sum \limits_{i=1}^n\left(\frac{\left|v-r\right|}{r}\right)\times 100 $$

where |*v* − *r*| represents the absolute difference between *v* and *r*.

The same method was used to determine whether fracture severity affected the skin surface temperature above vertebrae by stratifying groups into those with minor or major vertebral compression fractures (vertebrae D1 versus D2/3). The statistical data analysis package SPSS® version 22 was used for analysing the data. Paired *t* tests were performed to determine whether the skin temperatures above the fractured and healthy vertebrae were significantly different from their respective adjacent reference skin regions. Given the sample size, however, the outcomes of the *t* tests should be treated with caution.

## Results

Of the 11 patients recruited into the study, complete temperature measurements were obtained for eight patients and incomplete measurements for three. Incomplete measurements were obtained either because the fracture severity could not be determined, there were not any healthy vertebrae for comparison or there were no fractured vertebrae. The mean age of the patients was 11.8 years (standard deviation 4.0 years) and 63.6% (seven patients) were male. The patients’ demographics are provided in the Table 2.

**Table 2:** Patients’ demographics

Characteristic	OI participants (*n* = 11)
Weight (kg)	
Mean (SD)	37.9 (20.0)
Height (cm)	
Mean (SD)	136.1 (24.2)
BMI (kg/m^2^)	
Mean (SD)	18.9 (4.2)
OI type	
I	8 (72.7%)
II	0
III	1 (9.1%)
IV	2 (18.2%)
Participant position	
Sitting	10 (90.9%)
Lying on side	1 (9.1%)
Bisphosphonate treatment	
IV Pamidronate	6 (54.5%)
IV Zoledronic acid	3 (27.3%)
Oral Risedronate	2 (18.2%)
Room temperature	
Mean (SD)	24.19 °C (0.82)
Room relative humidity	
Mean (SD)	32% (5.17)

### Temperature percentage change between the skin surface above fractured thoracic vertebrae and skin reference regions

**Fig. 9:**
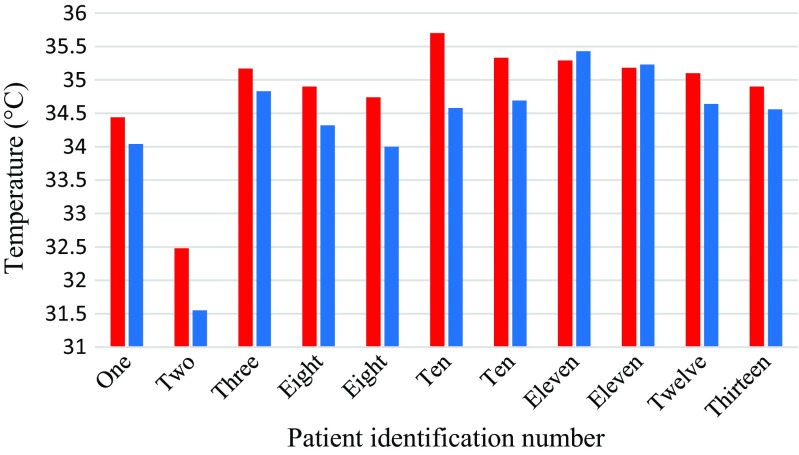
Bar chart showing mean temperatures of the skin above the fractured thoracic vertebrae (red) and their reference skin regions (blue)

The study mainly focused on the thoracic vertebrae as they accounted for the largest number of fractures. A summary of the temperatures for fractured thoracic vertebrae is provided in Table 3 and they are plotted in Fig. 9. The mean temperature differences between the skin surface above the fractured thoracic vertebrae and their adjacent reference skin regions assessed using TPC and ATPC were 1.44 (standard deviation 1.12) and 1.54 (standard deviation 0.97) respectively. The paired *t* test performed indicated that there was a significant difference between the mean temperatures of the skin surface over the fractured thoracic vertebrae and the reference skin regions (*p* = 0.002, confidence level = 95%).

**Table 3:** TPC and ATPC between the fractured thoracic vertebrae and their reference skin regions

Patient number	Fractured vertebrae	Temperature of fractured vertebra (°C)	Temperature of the reference skin region(°C)	TPC	ATPC
1	T8 to T12	34.44	34.04	1.18	1.18
2	T11 and T12	32.48	31.55	2.95	2.95
3	T11 and T12	35.17	34.83	0.98	0.98
8	T9	34.90	34.32	1.69	1.69
8	T11	34.74	34.00	2.18	2.18
10	T12	35.7	34.58	3.24	3.24
10	T7-T8	35.33	34.69	1.84	1.84
11	T6	35.29	35.43	− 0.40	0.40
11	T8 and T9	35.18	35.23	− 0.14	0.14
12	T6 and T7	35.10	34.64	1.33	1.33
13	T12	34.90	34.56	0.98	0.98
Mean		34.84	34.35	1.44	1.54
Standard deviation		0.85	1.03	1.12	0.97

### Temperature percentage change between healthy thoracic vertebrae and their skin reference regions

**Fig. 10:**
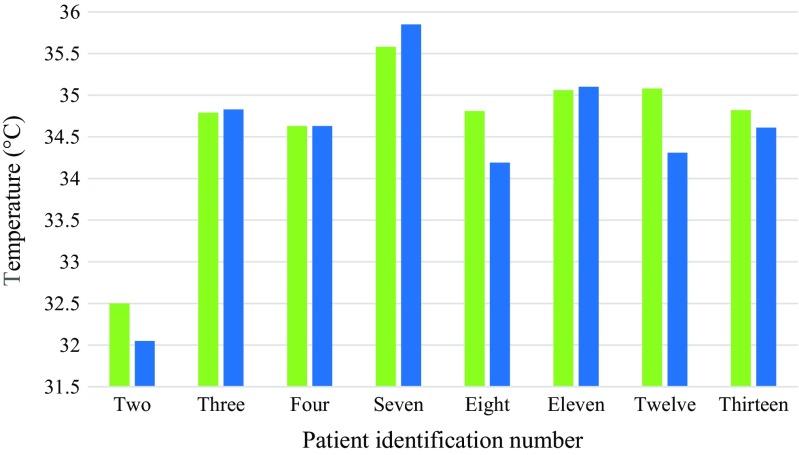
Bar chart showing average temperatures of the skin above the healthy thoracic vertebrae (green) and their reference skin regions (blue)

A summary of the temperatures for healthy thoracic vertebrae is provided in Table 4 and they are plotted in Fig. 10. The mean temperature differences between the skin surface above the healthy thoracic vertebrae and their adjacent reference skin regions assessed using TPC and ATPC were 0.97 (standard deviation 1.01) and 0.88 (standard deviation 0.82) respectively. The paired *t* test indicated that there was not a significant difference between the mean temperatures of the skin surface over the heathy thoracic vertebrae and their reference skin regions (*p* = 0.15, confidence level = 95%).

**Table 4:** TPC between healthy thoracic vertebrae and their reference regions

Patient number	Healthy vertebra	Temperature of healthy vertebra (°C)	Temperature of the reference skin region(°C)	TPC	ATPC
2	T5 to T7	32.50	32.05	1.40	1.40
3	T6 to T8	34.79	34.83	− 0.11	0.11
4	T4 to T5	34.63	34.63	0.00	0.00
7	T6 to T8	35.58	35.85	− 0.75	0.75
8	T10	34.81	34.19	1.81	1.81
11	T11	35.06	35.10	− 0.11	0.11
12	T11 to T12	35.08	34.31	2.24	2.24
13	T8	34.82	34.61	0.61	0.61
Mean		34.66	34.45	0.97	0.88
Standard deviation		0.92	1.10	1.01	0.82

**Fig. 11:**
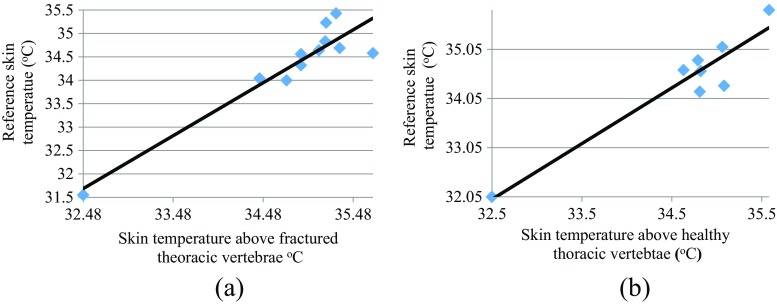
Correlation between the skin surface temperatures of the vertebrae and their related reference for **a** fractured thoracic vertebrae, **b** heathy thoracic vertebrae

Figure 11a, b shows the plots of skin surface temperatures of the reference region against the skin surface temperature above fractured and heathy thoracic vertebrae. The correlation coefficients for Fig.11a, b were 0.936 and 0.949 respectively. These indicate that the skin surface temperatures above these fractured and heathy vertebrae are correlated with their skin surface temperatures of their adjacent skin reference regions. The correlation for the case of the fractured vertebrae is reduced (as compared with the healthy vertebrae) but the high correlation could be because the percentage of temperature increase observed at the fractured sites is relatively small as compared with the actual skin temperature.

**Fig. 12:**
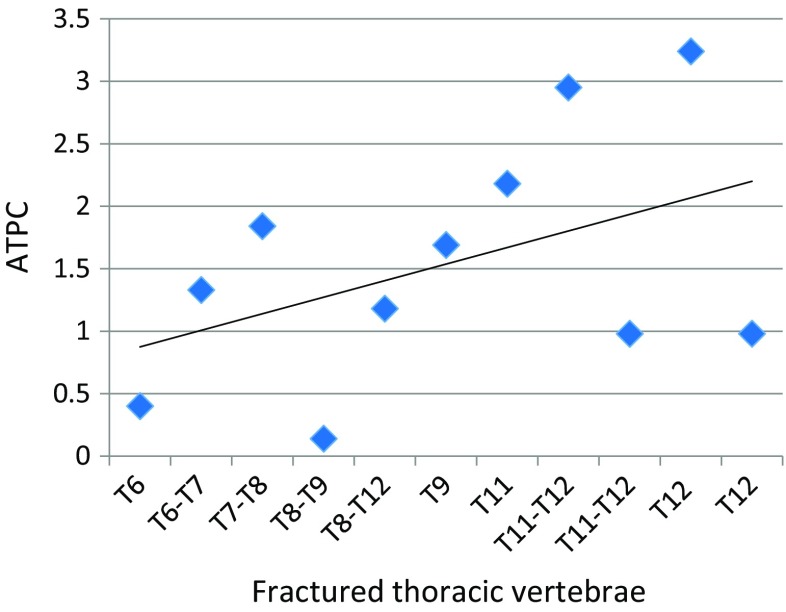
APTC for the thoracic vertebrae organised in relation to their positions on the spine

Figure 12 shows the plot of ATPC between fractured thoracic vertebrae and reference regions (Table 3) sorted in order of their positions on the spine, from the highest to lowest. The gradient for the best straight line through its points is 0.133. Although there is a significance scatter between the points and the best straight line, the trends represent an increase in APTC. This could be because the amount of adipose tissue surrounding the spine is greater amongst the lower thoracic and lumbar vertebrae than the upper thoracic vertebrae.

**Fig. 13:**
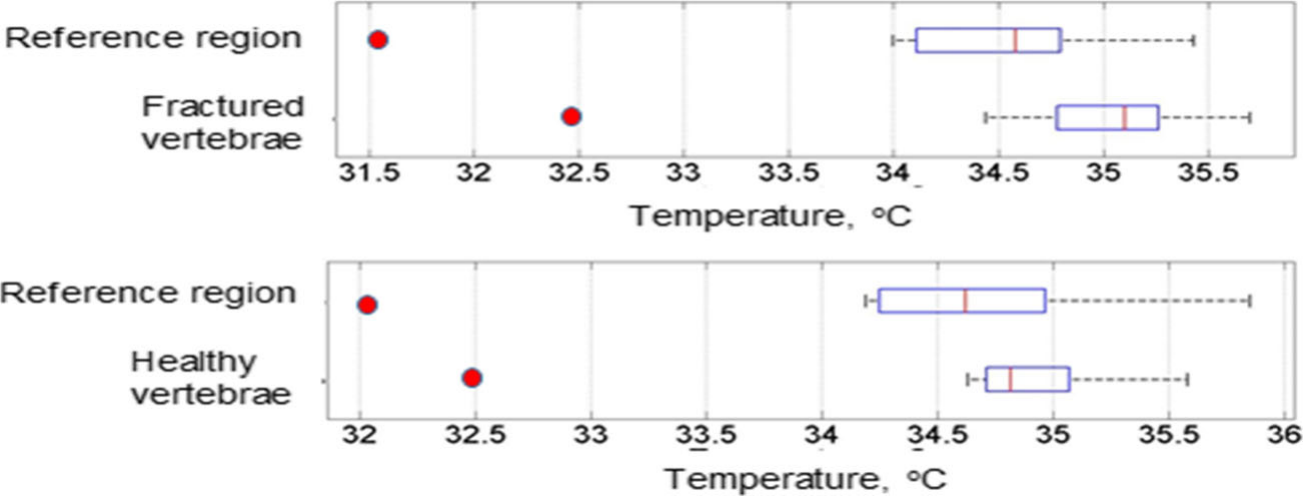
Box plots of temperatures of the fractured thoracic vertebrae and their related reference regions (top figure), temperatures of healthy thoracic vertebrae and their reference regions (bottom figure) (red points indicate outliers)

Figure 13 shows box plots of temperatures for the skin surface above the fractured thoracic vertebrae and their related adjacent reference regions (top part of the figure) and temperatures for the healthy thoracic vertebrae and their related adjacent reference regions (bottom part of the figure). The figure indicates that the medians of the temperatures of the fractured thoracic vertebrae and their reference regions are further apart as compared to those for the healthy thoracic vertebrae and their references. It also shows there is a greater temperature deviation for the fractured thoracic vertebrae and their reference regions as compared with the healthy thoracic vertebrae and their reference regions.

### Temperature percentage change between the skin surface above fractured thoracic vertebrae and healthy reference vertebrae

**Fig. 14:**
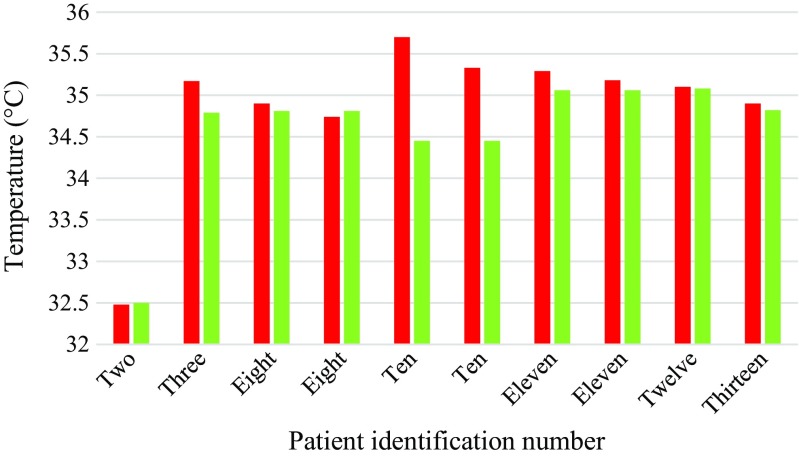
Bar chart showing mean temperatures of the skin above the fractured thoracic vertebrae (red) and their healthy reference vertebrae (green)

A summary of the temperatures for fractured thoracic vertebrae and their heathy reference vertebrae is provided in Table 5 and they are plotted in Fig. 14. The mean temperature differences between the skin surface above the fractured thoracic vertebrae and the skin surface above healthy vertebrae as assessed using TPC and ATPC were 0.86 and 0.92 respectively. Paired *t* test carried on the temperatures for the fractured thoracic and healthy reference vertebrae showed no significant difference (*p* = 0.0592, 95% confidence level). The fractured and their reference healthy vertebrae were at different levels in the spinal column due to fractures occurring in different parts of the spine and healthy vertebrae for comparison at the same levels were unavailable. Using healthy vertebrae as reference that are at different levels to the fractured vertebrae could be inappropriate as their positions may be affected by temperature. This observation could be consistent with the information provided in Fig. 12 where the trend for plot for *ATPC* has increased as position of thoracic vertebrae increased.

**Table 5:** TPC between fractured thoracic vertebrae and their healthy reference vertebrae

Patient number	Fractured vertebra	Temperature of fractured vertebra (°C)	Healthy vertebra	Temperature of healthy vertebrae (°C)	TPC	ATPC
2	T11 + T12	32.48	T5-T7	32.50	− 0.08	0.08
3	T11 + T12	35.17	T6-T8	34.79	1.10	1.10
8	T9	34.90	T10	34.81	0.27	0.27
8	T11	34.74	T10	34.81	− 0.21	0.21
10	T12	35.70	L4	34.45	3.66	3.66
10	T7-T8	35.33	L4	34.45	2.56	2.56
11	T6	35.29	T11	35.06	0.67	0.67
11	T8-T9	35.18	T11	35.06	0.34	0.34
12	T6-7	35.10	T11-T12	35.08	0.06	0.06
13	T12	34.90	T8	34.82	0.22	0.22
Mean		34.88		34.58	0.86	0.92
Standard deviation		0.89		0.76	1.27	1.22

### Temperature percentage change between D1 fractures and D2/3 fractures

The mean TPC between the skin surface over D2/3 fractured vertebrae and their skin reference regions was 1.57% (SD = 1.83); however, the number of cases considered for this test was insufficient to draw conclusions. The mean TPC between D1 fractured vertebrae and their skin reference regions was 1.14% (SD = 1.14, *p* = 0.01, 95% confidence level). There was a significant difference between the TPC of D1 fractured vertebrae and their reference regions (Table 6). The mean difference between the skin surface above D2/3 fractures and reference regions and the skin surface above D1 fractures and reference region was 0.43%. However, the number of cases was too small to draw conclusions from.

**Table 6:** TPC between DI and D2/D3 fractures

Statistical measure	TPC for D2 (*n* = 1) and D3 (*n* = 2) vertebrae and their skin reference regions	TPC for D1 (*n* = 9) vertebrae and their skin reference regions
Mean	1.57%	1.14%
SD	1.83	1.02
CI	(− 2.98, 6.12)	(0.36, 1.92)
*p* value	0.276	0.01

## Discussion

Children with OI can be subject to frequent ionising radiation to screen for vertebral fractures, and so, a non-ionising imaging technique that can assist in detection of fractures would be a beneficial tool. The use of high resolution thermal imaging (HRTI) to assess for vertebral fractures in children with OI has not been previously studied. Using HRTI and image processing, our group has developed a method which compares the temperature of the skin surface above vertebrae and the skin surface region adjacent to the selected vertebra. This technique could offer a potentially novel means of assisting the detection or monitoring of thoracic vertebral fractures in children with OI. The methodology had to be practical with regard to simplicity of operation to allow its applicability in routine medical diagnostic scenarios, reproducible and acceptable to patients. The methodology was based on the hypothesis that vertebral fractures may change the skin surface temperature above them and the associated temperature change could be detectable by HRTI. We observed a greater temperature percentage increase between the skin surface over the fractured thoracic vertebrae and their reference skin regions as compared to those for the healthy (not fractured) thoracic vertebrae and their associated reference regions.

Fracture severity was also considered to see whether this affected TPC. The mean TPC between the skin surface above D1 fractured vertebrae and the reference skin region was compared to those for D2 and D3 fractured vertebrae and reference region. The mean TPC between D2 and D3 vertebrae and reference region was 0.43% higher than between D1 vertebrae and their reference region and it was not significant. All patients had received bisphosphonate medication which would have helped their vertebrae regain height and could explain why there were fewer D2 and D3 fractures compared to D1 fractures. As the number of D2 and D3 vertebral fractures was quite small to determine whether fracture severity affects temperature, we suggest that a larger data set will be needed to explore this further. However, the greater temperature difference observed in D2 and D3 vertebrae suggests that more severe vertebral fractures may generate more heat.

The greater TPC observed in vertebral fractures may be due to the inflammatory effect of fracture healing. Increased blood flow to the area may also result from angiogenesis to transport osteoprogenitor cells for bone repair [19]. However, these mechanisms occur days to weeks after bone fracture and the vertebral fractures in these participants were likely to be at least 6 months old unless they had re-fractured in the same region. However, in children, vertebral remodelling at vertebral end plates continues to occur until they stop growing. This could explain the increase in temperature observed despite the fracture being old.

For patient in Table 3, the skin surface temperatures above the fractured vertebrae (T6, T8 and T9) were lower than the temperatures for their related adjacent reference skin regions (TPC values − 0.40 and − 0.14). These TPC values were the lowest amongst all patients. This patient has the highest adjacent reference skin temperatures (i.e. 35.43 and 35.23 °C). It is possible that the possible temperature increase at the site of fracture is masked by these higher reference regions’ temperatures. This might indicate that the possible temperature changes brought about by fractures may be detectable when the skin temperature is within a certain limit. This is an area of further exploration on a larger number of patients. All 11 patients included in the study were on bisphosphonate medication and they had varying degrees of vertebral fractures. This pilot study was not sufficiently detailed to explore the possible effects of medical treatment and fracture severity on the measured temperatures and these can be explored in future studies.

In three patients (i.e. patients 3, 7 and 11) indicated in Table 4, the temperatures above the healthy thoracic vertebrae were lower than their related adjacent reference skin regions (TPC values − 0.11, − 0.75 and − 0.11 respectively). The adjacent reference skin temperatures for these three patients were 34.83, 35.85 and 35.10 °C respectively. Patient 7 with TPC value of − 0.75 has the largest temperature for the reference skin region amongst all patients. As indicated in the previous paragraph, it may be possible that the temperature influence of vertebrae above skin surface may be accurately detectable if the skin temperature is within a certain bound.

The distance between the fractured vertebrae and its selected reference skin region is an area that needs further exploration. If the reference region is near the fractured vertebrae, its temperature can be influenced by the fracture, and if its distance is large, then the reference region does not properly represent the skin surface above the vertebra.

The temperature and humidity of the data recording room can influence the accuracy of the results. In this study, these parameters were measured and entered in the camera setting prior to the recordings to ensure camera was properly calibrated. Although the recording duration for this study was short, the temperature and humidity can slightly vary, and so for clinical diagnostic situations, care needs to be taken to control these as much as practical.

This was a pilot study with a number of limitations, the main one being the number of patients included the study. We have plan for a further study with a larger number of patients and a better exploration of the issues that could have influenced the accuracy of results.

We conducted the same analysis on fractured and healthy lumbar vertebrae (data not included) but we were unable to reproduce the findings in the lumbar region. Numbers of fractured vertebrae in our cohort were lower. Additionally, TPC may only be significant for thoracic vertebrae because of the large variation in skin thickness that occurs across the back. In the thoracic vertebrae and reference regions, the skin is thinner and there is little adipose tissue. This means the skin temperature recorded closely reflects its underlying structures whereas, in the lower back, there is more adipose tissue which, due to its insulating properties, alters the heat transfer between the core and skin and affects its thermal conductivity [20].

### Other potential causes of temperature change

**Fig. 15:**
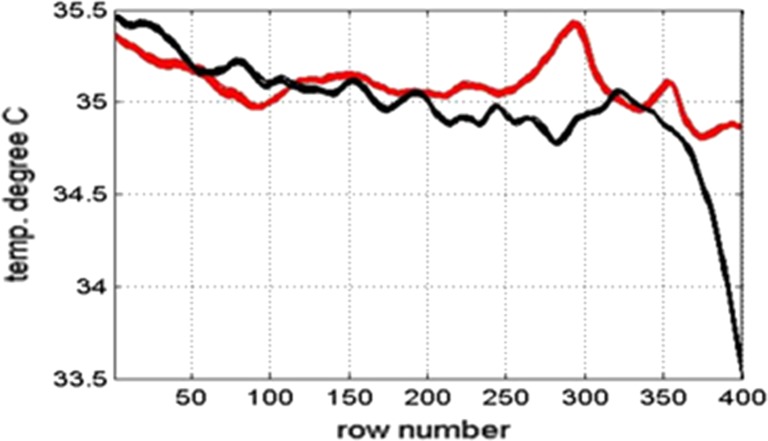
Plot of skin surface temperature over the whole skin (red) and plot of reference skin surface temperature next of the spine (black) for patient 11

The changes in temperature observed may not be caused by blood flow to the vertebra but from muscle or ligament damage near the vertebra. This is exemplified in patient 11. Figure 15 shows the mean temperature of the whole spine and its adjacent skin region for this patient.

There is a rise in temperature of almost 0.5 °C between image row numbers 270–310 (Fig. 15). Comparing the graph to the thermal and DXA images indicates this is at the level of the lumbar L2 vertebra. Patient 11 did not have any fractures in the lumbar region but complained of lower back pain when sitting at school so ligament or muscle damage could explain the pain and temperature peak. This is a possible confounding factor so should be considered in future studies.

Previous work using HRTI has shown promising results in identifying paediatric forearm fractures and also identified that larger sample sizes and more frequent measurements are needed [21].

The results from this study showed there was a significant difference in TPC between the skin surface above the fractured thoracic vertebrae and their adjacent reference skin regions and that the same may be true of lumbar vertebrae had a greater number of lumbar fractures been observed. However, the range of temperatures identified in fractured thoracic vertebrae between individual patients needs to be considered, and so, further work is required on a much larger cohort of OI patients to determine the validity and reproducibility of this novel methodology and to determine the clinical acceptability of the approach. Further work will also help to determine whether this methodology can be used to identify new fractures or to monitor fracture progression, once the fracture is initially identified by DXA or X-ray radiograph. The method could also be studied further to explore the medication effect on fracture recovery and provide more insight as to whether the technique is confined to the thoracic region or can be implemented for fracture detection throughout the spine.

## Conclusion

We have proposed a new methodology based on high resolution thermal imaging to identify thoracic vertebral fractures in children and young people with osteogenesis imperfecta (OI). The method was evaluated by obtaining thermal videos of 11 children and young people with OI to determine whether there was a temperature difference at known vertebral fracture sites compared to their adjacent skin regions.

This pilot study demonstrated the potential ability of HRTI to detect thoracic vertebral fractures. Initial results also point towards an association between thermal output and fracture severity although further work with a greater number of patients is required to verify this finding. This methodology may provide a potentially novel imaging method for fracture monitoring and fracture detection.
